# Tape-Worm Cured by Sp. Vin. Camph

**Published:** 1811-01

**Authors:** W. Goodwin


*To the Editors of the Medical and Physical Journal:*

*Tape-worm cured by Sp. Vin. Camph.*

GENTLEMEN,

IF the following accidental cure of a tape-worm, and the singular occurrence of a fair lady turning as black as a negro, appear worthy of your Journal, they are at your service.

W. GOODWIN.

A poor woman of Leeds, in Yorkshire, of the name of Weird, undertook to walk to the vicinity of Stowmarket in Suffolk, to see her daughter, who had married a militia man. The great length of the journey crippled and swelled her ancles so much, as to occasion her sending to a neighbouring surgeon for some spirits to bathe them with, and she received some spt. vin. camph. for that purpose. The old lady admired its look and smell, and having for two years past been afflicted with pain in her stomach, observed to her daughter, "what was gude for the *oote* side might be gude for the *in*, and that she would e'en take a gulp of it." Accordingly she drank about an ounce, and bathed her ancles with the remainder.

In about three hours after this, she felt something twisting itself about her thigh, and upon examination found a living tape-worm, upwards of three yards long; and from that time to this, now more than two years, has been entirely free from pain in her stomach. Her daughter has returned the visit, and found her mother in good health, with some of the "gude stuff" in the window, ready if the pain should return.

Since knowing of the above, a Mrs. Pipe applied to me on account of a bilious, nervous affection, with loss of appetite, &c. for which she had medicines and advice; but not getting quite well, observed she had forgot to mention a strange unpleasant sensation in her stomach, attended with slight pain at times, and a rising up to her throat, as of something alive; and saying also, that a long worm had lately crept into her mouth, I advised her to take a glass of gin, with two tea-spoonsful of spt. vin. camph. in a morning fasting, which she readily did, and from the time of doing so, lost all the distressing sensation in her stomach, has recovered her health and appetite, and been well ever since, now several months. She has no doubt but the worms in the stomach were the cause of her ill health, and firmly believes the spirits killed them; but as she visited the temple in the garden, no examination for the vermes was thought of.

Mrs. E———, a single woman, about 60 years of age, till about 20 years since, was of a natural fair complexion; when having an illness of some continuance, she perceived on her recovery that her complexion had changed to a dark hue, which has since gradually increased to that of the darkest native of Africa.

Mrs. E. is in general good health, but occasionally complains of rheumatism. I should be glad if some of your correspondents would favour us with their opinions of the cause of this singular phenomenon.

